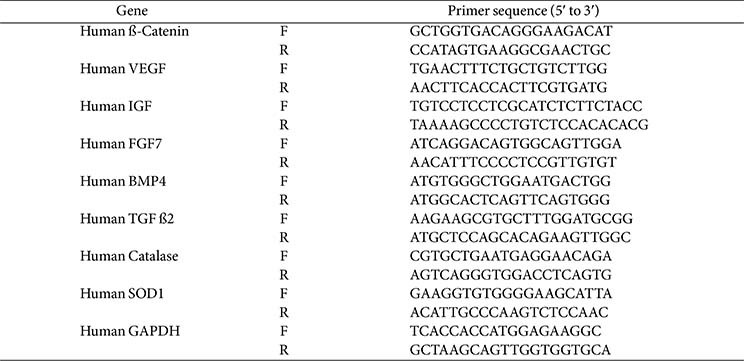# Pea Sprout Extract Promotes Hair Follicle Regeneration via Anagen Phase Prolongation and Dual Modulation of Oxidative and Inflammatory Signaling

**DOI:** 10.4014/jmb.2508.08011

**Published:** 2025-11-26

**Authors:** Yujin Kim, So Hyeon You, Daewon Yoon, Jung Min Lee, Mi Seon Woo, Yea Ji Park, Jun-Pyo Hong, Joon Seok, Beom Joon Kim

**Affiliations:** 1 Department of Dermatology, College of Medicine, Chung-Ang University, Seoul 06974, Republic of Korea; 2 Department of Medicine, Graduate School, Chung-Ang University, Seoul 06973, Republic of Korea; 3 CH Labs Corp., Seoul 04300, Republic of Korea; 4 Chong Kun Dang Healthcare, Seoul 04300, Republic of Korea; 5 Fine BS Co., Ltd., 76 Yeonmujang-gil, Seongdong-gu, Seoul 04784, Republic of Korea

**Keywords:** Pea sprouts extract, hair loss, Dermal papilla cells (DPCs), Wnt/β-catenin, anti-oxidation

## Abstract

Hair loss is a prevalent condition that affects individuals across all ages and genders. Its causes are multifactorial, involving genetic predisposition, hormonal imbalances, aging, stress and air pollution factors. A previous randomized clinical trial, reveals that daily administration of 100mg of pea sprout extract (PSE) for 8 weeks significantly increased hair density in patients, however, the underlying mechanisms remained unclear. Therefore, this study aims to investigate the efficacy and underlying mechanisms of PSE in promoting hair growth. *In vitro* experiments were conducted using human dermal papilla cells (hDPCs) and RAW 264.7 macrophage cells alongside an *in vivo* study in a C57BL/6 mouse model. Our study demonstrates that PSE promotes hair growth by activating the Wnt/β-catenin signaling pathway, upregulating hair growth-promoting factors, including IGF-1 and FGF7, while downregulating growth inhibitory factors, including IL-1β and BMP4. This indicates that PSE effectively modulates the balance of growth factors by enhancing dermal papilla-associated signaling, thereby harmonizing the hair growth cycle. Furthermore, our results reveal that PSE exhibits anti-oxidant and anti-inflammatory properties, which may help protect hair follicles from oxidative stress and inflammatory damage, processes that can be influenced by environmental pollutants. Inflammation relief involves the use of anti-inflammatory agents to suppress pro inflammatory cytokines, thereby improving the microenvironment surrounding hair follicles and supporting hair growth. PSE is a promising natural ingredient, offering potential benefits in combating hair loss and promoting healthier hair growth by modulating key signaling pathways and providing protective effects. Its potential as a potential natural alopecia treatment warrants more research.

## Introduction

Alopecia is a significant condition that significantly impairs physical health and quality of life [1]. Furthermore, it is not limited to a specific age groups or gender, impacting individuals of all ages [2, 3]. Alopecia is a complex condition resulting from the interplay of various factors, including genetic susceptibility, hormonal changes, stress, immune dysregulation, and lifestyle factors [4]. While genetic predisposition remains a principal factor in hair loss, recent studies increasingly implicate environmental exposures and psychological stress as significant contributor [2, 5]. Accumulation of dust and impurities on the scalp can obstruct hair follicles and triggers localized inflammatory responses [6, 7]. These responses include the activation of immune cells and the release of pro-inflammatory cytokines, such as interleukin-1 beta (IL-1β) and tumor necrosis factor-alpha (TNF-α) [8]. Pro-inflammatory cytokines promote apoptosis in hair follicle cells, ultimately leading to damage and impaired the hair shaft growth [9]. In parallel, reactive oxygen species (ROS) generate oxidative stress within the scalp microenvironment. This oxidative stress is implicated in the induction of apoptosis in hair follicle cells, thereby contributing to hair loss [10]. To counteract the harmful effects of ROS, the activation of endogenous antioxidant enzymes such as superoxide dismutase (SOD) and catalase (CAT) is crucial to scavenge or neutralize these harmful species [11]. Maintaining a balance between ROS generation and antioxidant defense mechanisms is critical for preserving hair follicle integrity under stress conditions.

Hair growth and regression are mediated by the hair cycle, a precisely regulated process comprising three distinct phases: anagen, catagen, and telogen [12]. During the anagen phase, active hair growth. At the base of the hair follicle, the dermal papilla, which consists of dermal papilla cells (DPCs), plays a pivotal role in hair cycle progression [2]. DPCs secrete various growth factors, including insulin-like growth factor (IGF-1), fibroblast growth factor 7 (FGF7), vascular endothelial growth factor (VEGF), and epidermal growth factor (EGF), that stimulate hair follicles by promoting the proliferation and differentiation of hair-growth–related cells, primarily via activation of pathways such as Wnt/β-catenin [13]. The catagen phase represents a transitional period in which hair follicle activity slows and production ceases. This transition from anagen to catagen is partly regulated by inhibitory factors secreted by DPCs, including bone morphogenetic protein 4 (BMP4) and transforming growth factor beta 2 (TGF-β2). Catagen phase is followed by telogen phase, a resting phase characterized by arrested follicular activity, and eventual hair shedding [14]. Under normal physiological conditions, the hair cycle maintains a typical anagen to telogen ratio of approximately 9:1. However, disruption of this cyclical balance, particularly an increase in the proportion of follicles in the telogen phase exceeding 25% and accompanied by daily hair shedding exceeding 100 hairs, is clinically recognized as alopecia [15].

Finasteride and minoxidil remain the only pharmacological agents approved by the United States Food and Drug Administration (FDA) for hair loss treatment, however, both exhibit significant limitations [16]. Minoxidil is applied topically to the scalp and promotes hair growth by enhancing blood flow through vasodilation [17]. However, its formulation can lead to undesirable effect such as stickiness and scalp irritation [18]. Finasteride is an oral medication that inhibits dihydrotestosterone (DHT), a key androgen involved in the pathogenesis of androgenetic alopecia, thereby preventing hair loss [19]. However, its use has been associated with adverse effects such as sexual dysfunction and an enhanced risk of depressive symptoms [20]. Furthermore, the treatment is not curative, as hair loss typically recurs upon discontinuation [21].

PSE is widely recognized for its nutritional value, established safety and common dietary use. It contains various essential nutrients, high levels of protein, minerals such as potassium, phosphorus, vitamin E, and various amino acids [22]. Clinical studies reports that PSE effectively inhibits hair loss [23]. Moreover, pea sprouts are known to be rich in phenolic compounds and flavonoids, which contribute to their antioxidant and anti-inflammatory effects [24, 25]. However, the precise underlying mechanisms and specific factors through which it exerts these effects remain unclear and warrant further investigation. Therefore, this study aims to investigate the hair growth-promoting activity of PSE using *in vitro* and *in vivo* models.

## Materials and Methods

### Pea Sprout Extract Preparation and Reagent

PSE, represented by AnaGain Nu, was utilized to evaluate its effects on hair growth. Developed by Mibelle AG, Switzerland, AnaGain Nu was supplied in powder form by Fine BS Co., Ltd., and CH Labs Co., Ltd., both based in Republic of Korea. Prior to use, the powder was dissolved in distilled water and filtered through a syringe filter. Furthermore, the specific batch of AnaGain Nu employed in the current research is certified by Ecocert Greenlife according to the COSMOS standard, demonstrating its compliance with rigorous criteria for natural and organic cosmetics and supporting its designation as a safe ingredient.

Minoxidil (MNX) was purchased from Sigma-Aldrich (USA). Antibodies specific to Wnt3a, β-catenin, GSK3β, TNF-α, IL-6, and β-actin were purchased from Cell Signaling Technology Inc. (USA). IL-1β was purchased from Affinity biosciences (USA). IGF1, FGF7, BMP4, VEGF and TGFβ2 were purchased from Invitrogen (USA).

### Cell Culture

The human follicle dermal papilla cells(hDPCs) used to evaluate hair growth and antioxidant effects were obtained from Cefobio (Republic of Korea). The culture medium for hDPCs consisted of dulbecco’s modified eagle’s medium (DMEM; Gibco, USA), supplemented with 10% fetal bovine serum (FBS; Invitrogen, USA) and 1% penicillin (100 IU/ml) and streptomycin (100 μg/ml) (PS; Invitrogen). RAW 264.7 used to evaluate anti-inflammatory effects were obtained from korean cell line bank (KCLB; Republic of Korea). The growth medium for RAW 264.7 cells was prepared using DMEM (Gibco) supplemented with 10% FBS and PS (Invitrogen). Both cell types were cultured in a humidified incubator at 37°C with 5% CO_2_ and were subculture every two days.

### WST-8 Assay

hDPCs were seeded into 96-well plates (Corning, USA) at a density of 1 × 10^4^ cells/well and incubated for 24 h to reach a confluency of 80%. After incubation, PSE was administrated at concentrations of 0, 1, 5, 10, 50, or 100 μg/ml, while MNX at 1 μg/ml, serving as the positive control, was treated as for 24 h. To evaluate cell proliferation, a WST-8 assay kit was used. Specifically, 10 μl of WST-8 solution was added to each well and incubated for 2 h. Absorbance was measured at 450 nm using a microplate spectrophotometer (SpectraMax 340; Molecular Devices, Inc., USA). The experiment was performed in 3 times, and mean cell viability was calculated to assess proliferation. The results are presented as a percentage relative to the control group.

### Anti-Oxidant Effects

hDPCs were co-treated with 150 μM H_2_O_2_ and PSE to investigate the cytoprotective effect of PSE against oxidative stress, followed by incubation for 24 h. After treatment, cell proliferation and the expression levels of antioxidant-related factors were evaluated.

### Anti-Inflammatory Effects

RAW 264.7 cells were co-treated with 1 μg/ml lipopolysaccharide (LPS) and PSE to evaluate the anti-inflammatory effects of PSE, followed by incubation for 24 h. After treatment, cell proliferation and the expression levels of inflammation-related factors were assessed.

### Reverse Transcription Followed by Quantitative Polymerase Chain Reaction (qPCR)

hDPCs and RAW264.7 cells were seeded into 6-well plates at a density of 1 × 10^6^ cells/well and incubated for 24 h. After incubation, cells were treated with the appropriate substances according to the experimental purpose and further incubated for an additional 24 h. Total RNA was extracted using TRIzol reagent (Invitrogen). RNA concentration and purity were measured using a NanoDrop spectrophotometer (Thermo Scientific, USA) with 2 μg of RNA. cDNA was synthesized using a cDNA Synthesis Kit (Thermo Scientific). qPCR was performed using SYBR Green Master Mix (Applied Biosystems, USA) on a LightCycler 480 System (Roche, USA). Gene expression results were calculated and reported as cycle threshold (Ct) values using the ΔCt quantification method. GAPDH was used for the normalization. The primer sequences are summarized in Table 1.

### Hair Regeneration Model

C57BL/6 male mice (6 weeks old) were purchased from Saeron Bio Inc. (Republic of Korea) and allowed to acclimatized for 1 week. The mice were housed under controlled conditions at 23 ± 2°C, with 50 ± 10% humidity, and a 12-h light/12-h dark cycle. All animal procedures complied with the NIH Guidelines for the Care and Use of Laboratory Animals and were approved by the Chung-Ang University Institutional Animal Care and Use Committee (IACUC No. A2022045). C57BL/6 mice transitioning from the telogen to anagen phase were prepared by shaving the dorsal skin during the telogen phase of the hair cycle, as previously described [26]. The mice were randomly assigned to four groups: Vehicle, PSE 25 mg/kg (mpk), PSE 50 mpk, and MNX 1 mpk. After imaging at day 0, one mouse was found deceased. Consequently, one mouse was removed from all groups, and subsequent experiments were conducted with 5 mice per group. All statistical analyses were performed using these 5 mice. PSE and MNX were administered orally five times per week for 14 days. To compare hair growth rates, the dorsal skin was photographed using a digital camera on days 0, 9, and 14 following depilation. The growth area per total area was calculated using ImageJ (version 1.52a NIH, USA). On day 14, the mice were sacrificed and dorsal skin tissues were collected for histological analysis.

### Western Blot Analysis

Following the indicated treatments, hDPCs were lysed using RIPA buffer (Thermo Fisher Scientific) to extract protein. Equal amounts of protein were separated by electrophoresis on 10% sodium dodecyl sulfate-polyacrylamide (SDS-PAGE) gels and subsequently transferred onto nitrocellulose membranes (Cytiva, USA). The membranes were blocked with 5% skim milk in Tris-buffered saline (TBS) containing 0.1% Tween-20 (TBS-T) and incubated with primary antibodies overnight at 4°C. Subsequently, the membranes were washed and incubated with HRP-conjugated anti-mouse or anti-rabbit secondary antibodies (Vector Laboratories Inc., USA). Immunodetection was performed using the Amersham ECL kit (GE Healthcare, USA) according to the manufacturer’s protocol. Protein bands were visualized using the ChemiDoc MP Imaging System (Bio-Rad Laboratories, Inc., USA) and analyzed with Image J software (NIH).

### Histology and Immunohistochemistry (IHC)

Dorsal skin tissues from each mouse were fixed with 10% formalin and embedded in paraffin blocks. The paraffin blocks were sectioned transversely or longitudinally and mounted onto glass slides. The slides were stained with H&E to evaluate the number of hair follicles (HFs), skin thickness, and the anagen/telogen ratio. The slides were observed using an optical microscope (Leica, Germany). For IHC, the tissue sections were immune stained with specific antibodies, including anti-BMP4, IGF-1, FGF7, and TGF-β (1:100, Invitrogen). The stained tissue slides were imaged using a slide scanner (Pannoramic MIDI; 3DHISTECH Ltd, Hungary) and observed using Case Viewer software. Hair follicle counts were performed on cropped images within a fixed area (1 × 1 mm). All histological examinations were analyzed in three sections per tissue sample from each mouse.

### ELISA

Blood samples were collected from mice on day 14 following drug administration. To obtain serum/plasma, the collected blood was centrifuged at 13,000 rpm for 15 min at 4°C, and the supernatant was carefully collected. IGF-1 concentrations in the blood samples were measured using a commercially available ELISA kit (Invitrogen). The assay was performed strictly according to the manufacturer's protocol. Absorbance was measured at 450nm using a microplate reader, and IGF-1 concentrations were calculated based on a standard curve generated from known IGF-1 standard.

### Statistical Analysis

The results are expressed as the mean ± standard deviation (SD) of at least three independent experiments. Statistical analyzed using one-way analysis of variance (ANOVA), followed by Tukey’s post hoc test. All statistical analyses were performed using GraphPad Prism 7.0 (GraphPad Software Inc., USA). Differences with a P-value of lower than 0.05 were considered statistically significant and indicated on the graphs with the following symbols: *, *P* < 0.05, **, *P* < 0.01, ***, *P* < 0.001; and ****, *P* < 0.0001.

## Results

### PSE Stimulates the Proliferation and Hair Growth-Related Gene Expression of hDPC

**Fig. 1:**
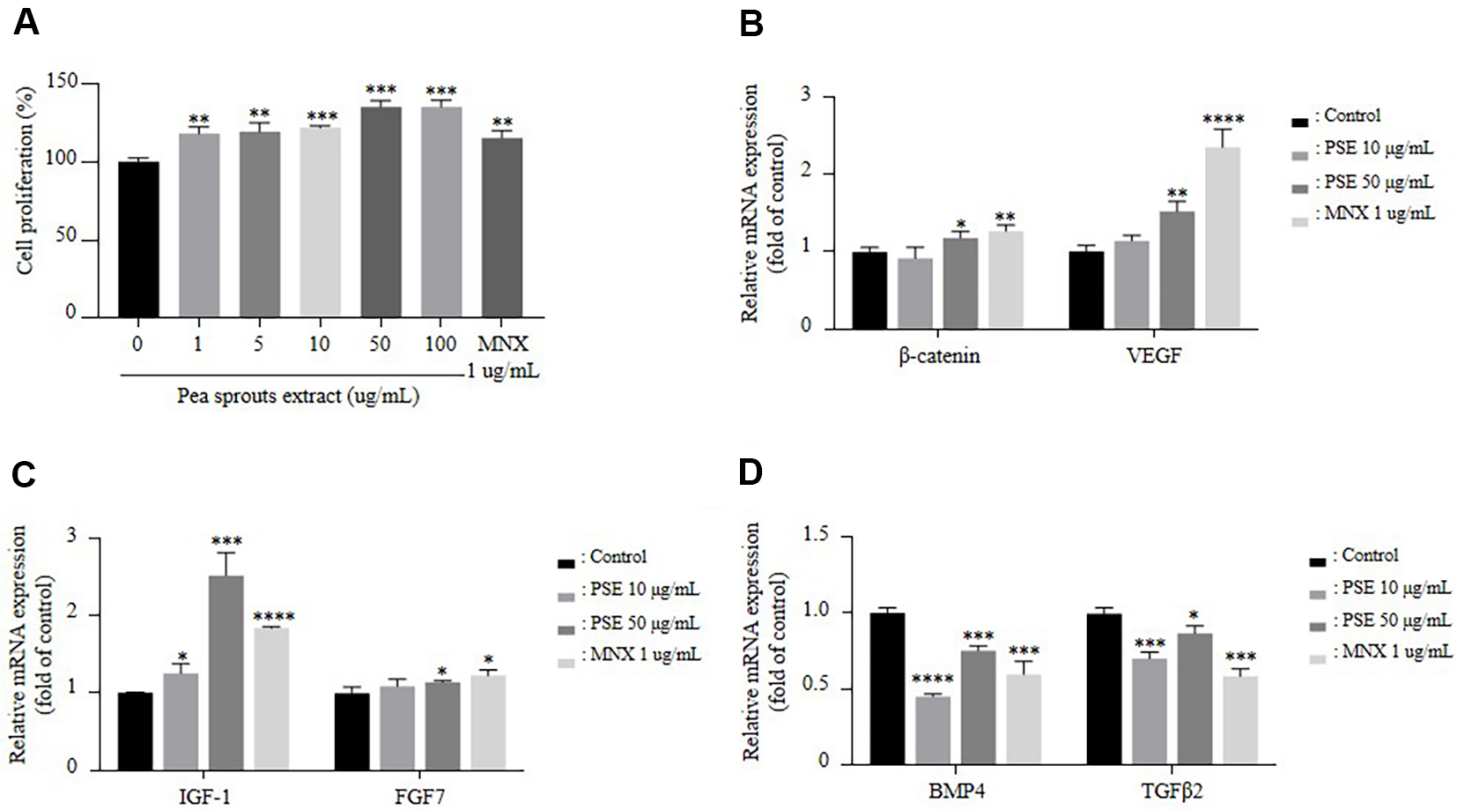
Pea sprout extract stimulates the hair growth-related gene expression. Cellular and molecular responses of hDPCs after 24-h treatment with PSE and MNX. (**A**) Viability of the hDPCs was measured after treatments with MNX (1 μg/ml) and PSE (10 and 50 μg/ml) using the WST-8 assay. (**B**) Relative mRNA expression levels of β-catenin pathway–related genes including β-catenin, and VEGF were measured after treatments with MNX (1 μg/ml) and PSE (10, 50 μg/ml) using a qPCR. (**C**) Relative mRNA expression levels of hair growth–promoting factors including IGF-1 and FGF7 were treated with MNX (1 ug/ml) and PSE (10, 50 μg/ml) using a qPCR. (**D**) Relative mRNA expression levels of hair growth–inhibiting factors such as BMP4, and TGFβ2 were treated with MNX (1 ug/ml) and PSE (10, 50 μg/ml) using a qPCR. *n* = 3. The results are presented as the mean ± SD. Statistical analyses were performed using one-way ANOVA followed by Tukey’s post hoc test. *P < 0.05, ** *P* < 0.01, *** *P* < 0.001, **** *P* < 0.0001 vs Control.

hDPCs constitute the dermal papilla located at the base of the hair follicle, which plays a crucial role in regulating hair growth and degeneration [27]. To investigate the effects of PSE, hDPCs were treated for 24 h with PSE at concentrations ranging from 1 μg/ml to 100 μg/ml or with MNX 1 μg/ml, a well-known hair growth-promoting agent. Cell proliferation was assessed using the WST-8 assay. Treatment with PSE resulted in a concentration-dependent increase in hDPC proliferation (Fig. 1A). β-catenin plays a pivotal role the regulation of hair growth [28]. The proliferation of hDPCs is regulated by various growth factors. Among these, IGF-1, VEGF, and FGF7, promote hair growth, whereas BMP-4 and TGF-β2 inhibit hair growth. qPCR analysis revealed that PSE treatment upregulated the expression of β-catenin, IGF-1, VEGF, and FGF7 while downregulating the expression of BMP-4 and TGF-β2 in hDPCs (Fig. 1B-1D).

### PSE Regulates Oxidative Stress and Inflammatory Responses

**Fig. 2:**
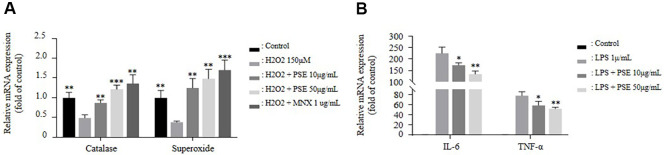
Pea sprouts extract regulates oxidative stress and inflammatory responses. (**A**) Relative mRNA expression levels of antioxidant enzymes including catalase and superoxide were measured using qPCR in hDPCs following 24-h treatment with H_2_O_2_ alone, or in combination with MNX (1 μg/ml) or PSE (10 and 50 μg/ml). (**B**) Relative mRNA expression levels of inflammatory cytokines including IL-6, and TNF-α were assessed using qPCR in RAW 264.7 cells following 24-h treatment with LPS alone, or in combination with MNX (1 μg/ml) or PSE (10, 50 μg/ml). *n* = 3. The results are presented as the mean ± SD. Statistical analyses were performed using one-way ANOVA followed by Tukey’s post hoc test. * *P* < 0.05; ** *P* < 0.01; ***P < 0.001; vs H_2_O_2_ or LPS.

Oxidative stress can induce apoptosis in hair follicles, ultimately contributing to hair loss. The activation of antioxidant enzymes can suppress the production of ROS, thereby reducing oxidative stress [29]. Furthermore, immune responses targeting hair follicles can trigger inflammation, resulting in hair follicle damage [30]. Compared to that of the group treated with H_2_O_2_ alone, co-treatment with PSE and H_2_O_2_ resulted in a dose-dependent increase in the expression of antioxidant enzymes, including catalase and SOD (Fig. 2A). Similarly, compared to that of the group treated with LPS alone, co-treatment with PSE and LPS resulted in a dose-dependent reduction in the elevated levels of IL-6 and TNF-α (Fig. 2B). These results suggest that PSE may reduce hair follicle damage by alleviating oxidative stress and suppressing inflammatory responses.

### PSE Induced Visible Hair Growth in C57BL/6 Mice

**Fig. 3:**
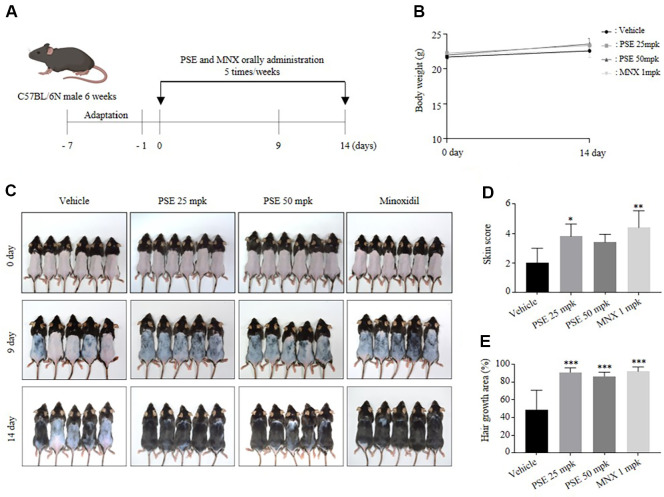
Pea sprout extract visually stimulates the hair growth of the C57bL/6 mice. The vehicle, PSE (25, or 50 mpk), or MNX (1 mpk) were orally administrated 5 times per week for 14 days. (**A**) Experiment scheme. (**B**) Body weight changes in mice over 14 days. (**C**) Photographs of the dorsal skin of mice taken with a DSLR camera. At day 0, *n* = 6. At day 9 and 14, *n* = 5. (**D**) Skin score measured on day 9. (**E**) Hair growth area measured on day 14. The results are presented as the mean ± SD. Statistical analyses were performed using one-way ANOVA followed by Tukey’s post hoc test. *P < 0.05, **P < 0.01, ***P < 0.001 vs vehicle.

Six-week-old mice were acclimated for one week before undergoing dorsal depilation. Starting the day after depilation, saline (vehicle group), PSE at 25 mpk, PSE at 50 mpk, and MNX at 1 mpk were orally administered at 100 μl per dose, 5 times per week for 14 days (Fig. 3A). On day 9, visual observation and skin score analysis revealed that the MNX group showed the most significant change in skin color, followed by the PSE 25 and 50 mpk groups, which exhibited more rapid changes in skin color compared to the vehicle group (Fig. 3C and 3D). The skin score is determined according to a previously described method [31]. On day 14, visual observation and quantitative analysis reveal that the MNX group exhibited the greatest hair growth, followed by the PSE 25 mpk and 50 mpk groups, all of which showed over 80% hair regrowth area. In contrast, the vehicle group exhibited approximately 50% hair growth (Fig. 3C and 3E).

### PSE Prolonged the Anagen Phase in C57BL/6 Mice

**Fig. 4:**
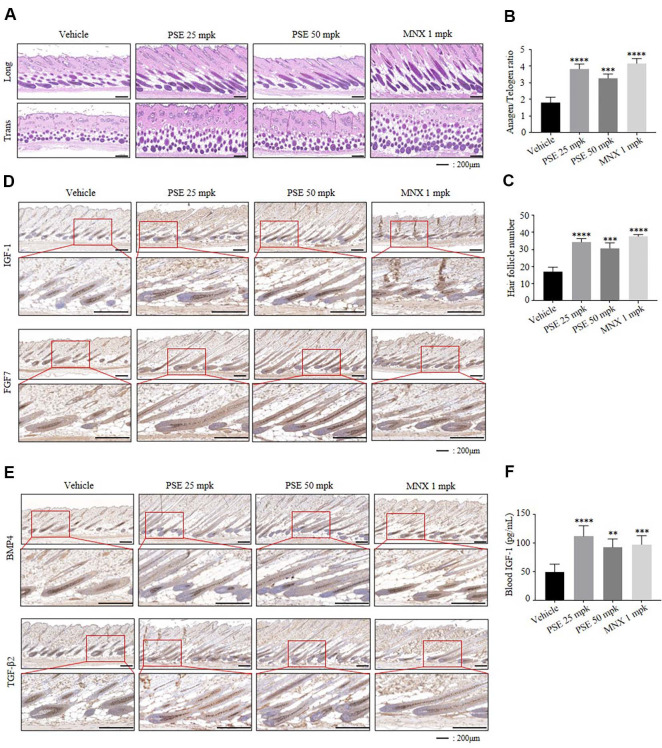
Pea sprout extract prolonged the anagen phase in C57BL/6 mice. The vehicle, PSE (25, or 50 mpk), or MNX (1 mpk) were orally administrated 5 times per week for 14 days. (**A**) Representative H&E-stained images of longitudinal and transverse sections of dorsal skin collected on day 14. (**B**) Anagen/telogen ratio measured from H&E-stained longitudinal sections on day 14. (**C**) Hair follicle count measured from H&E-stained transverse sections on day 14. (**D**) IHC analysis of hair growth–related growth factors including IGF-1 and FGF7 in skin tissue on day 14. (**E**) IHC analysis of hair growth–inhibiting factors including BMP4 and TGF-β2 in skin tissue on day 14. (**F**) Expression of IGF-1 in mouse serum on day 14 using ELISA. *n* = 3, The results are presented as the mean ± SD. Statistical analyses were performed using one-way ANOVA followed by Tukey’s post hoc test. **P < 0.01, ***P < 0.001, ****P < 0.0001 vs vehicle.

Among the growth factors that play key roles in hair growth, IGF promotes the prolongation of the anagen phase, FGF7 contributes hair shaft elongation, BMP4 inhibits anagen, and TGF-β2 induces the transition from anagen to catagen. On day 14, dorsal skin tissues were harvested to evaluate the condition of hair follicles, and tissue sections were prepared for H&E staining and immunohistochemical analysis of relevant markers. Longitudinal H&E-stained sections indicate that PSE increase the proportion of hair follicles in the anagen phase (Fig. 4A and 4B). While transverse sections show that PSE increase the number of hair follicles (Fig. 4A and 4C). In addition, PSE upregulates the expression of IGF-1 and FGF7, while downregulating BMP4 and TGF-β2 (Fig. 4D and 4E). Furthermore, when IGF-1 expression was examined in the blood of mice, PSE was found to increase its expression (Fig. 4F). Collectively these results show that PSE promote anagen transition, elongate anagen phase, and induce hair regeneration.

### PSE Enhance the Wnt/β-Catenin Signaling Pathway and Have Anti-Inflammation Effect

**Fig. 5:**
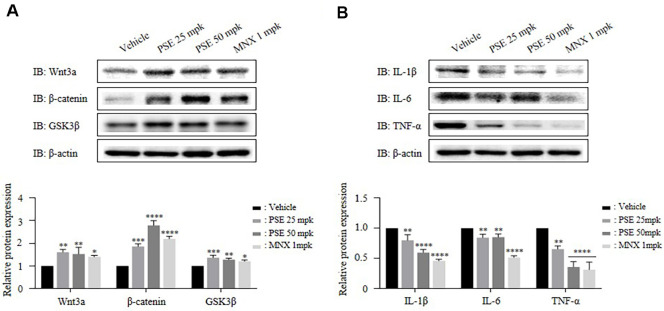
Pea sprout extract enhances the Wnt/β-catenin signaling pathway and exhibits anti-inflammation in mouse model. The vehicle, PSE (25, or 50 mpk), or MNX (1 mpk) were orally administrated 5 times per week for 14 days. (**A**) Protein expression of Wnt/β-catenin pathway–related factors including Wnt3a, β-catenin, and GSK3β using western blotting. (**B**) Protein expression of pro-inflammatory cytokines such as IL-1β, IL-6, and TNF-α using western blotting. *n* = 3, The results are presented as the mean ± SD. Statistical analyses were performed using one-way ANOVA followed by Tukey’s post hoc test. *P < 0.05, **P < 0.01, ***P < 0.001, ****P < 0.0001 vs vehicle.

The Wnt/β-catenin signaling pathway plays a critical role in regulating hair growth and regeneration [32]. The protein expression levels of Wnt3a, β-catenin, and GSK3β were significantly increased in the dorsal skins tissues of mice treated with PSE at 25 mpk, PSE at 50 mpk, and MNX (Fig. 5A). Inflammation within hair follicles contributes to follicular damage and can impair hair growth. The expression of pro-inflammatory cytokines such as IL-1β, IL-6, and TNF-α was reduced in skin protein extracts following treatment with PSE 25 mpk, PSE 50 mpk, and MNX.

## Discussion

This study investigates the molecular mechanisms underlying the clinically observed hair growth-promoting effects of pea sprouts extract (PSE). Clinical trials show that daily oral supplementation with 100 mg of PSE for 8 weeks significantly increases hair density [33]. However, the specific mechanisms underlying these effects remain largely unknown. To explore the molecular mechanisms, we used *in vitro* models (hDPCs and RAW 264.7 cells) and an *in vivo* C57BL/6 mouse model.

PSE treatment promoted the cell proliferation of hDPCs and *in vivo*, oral administration of PSE for 14 days accelerated anagen entry and enhanced hair growth compared to controls (Figs.1A and 3C-3E). Furthermore, histological analysis of mouse skin revealed an increased proportion of hair follicles in the anagen phase as well as a significant rise in the total number of follicles (Fig. 4A-4C).

Besides the mechanistic findings, *in vivo* mouse studies reveal a significant increase in the proportion of hair follicles in the anagen phase and total hair follicle numbers following PSE administration (Fig. 4A-4C). These dual effects likely act synergistically to prolong the anagen phase of the hair cycle, thereby enhancing overall hair growth. For example, the observed increase in both anagen follicle ratio and total follicle count in our mouse model provides strong supporting evidence for the increased hair density reported in clinical studies [33].

Consistent with this phenotype, both *in vitro* and *in vivo* models showed activation of the Wnt/β-catenin signaling pathway (Figs. 1B and 5A), which is central to the telogen-to-anagen transition [28, 32]. Also, PSE treatment led to elevated expression of growth factors such as IGF-1 and FGF7, while simultaneously suppressing the expression of inhibitory factors including BMP4 and TGF-β2 in both models (Figs. 1C, 1D, and 4D-4F). These changes align with Wnt/ β-catenin pathway activation, which enhances the secretion of pro-anagen growth factors [34, 35], and suppresses catagen inducing factors such as BMP4 and TGF-β [8, 36]. These molecular changes are likely to underlie the accelerated anagen entry and enhanced follicle growth observed. Our findings suggest that the Wnt/β-catenin pathway mediates the upregulation of growth factors; however, the precise transcriptional regulation by nuclear β-catenin has not been elucidated, and functional inhibition experiments were not performed. Therefore, future studies should validate this mechanism using Wnt antagonists and further confirm it in ex vivo models to more comprehensively elucidate the pathway by which PSE promotes hair growth.

Furthermore, PSE possesses antioxidant and anti-inflammatory effects, which may enhance its hair growth-promoting effects by protecting hair follicles from damage [37]. This supports the concept of an antioxidant scalp shield, providing a protective barrier against oxidative stress. Specifically, PSE showed antioxidant activity *in vitro* (Fig. 2A), and anti-inflammatory effects in both *in vitro* and *in vivo* models (Figs. 2B and 5B), likely due to the complex composition of Pea sprouts, the source material of PSE [38]. To clarify the underlying mechanism, further studies are needed to identify the specific bioactive compounds responsible for the observed antioxidant and anti-inflammatory effects. While these components likely contribute to overall efficacy, isolating the key compounds or synergistic combinations that primarily drive Wnt/β-catenin activation and hair growth promotion remains an important area for future research. Identifying these active principles could further optimize PSE's therapeutic potential. However, demonstrating the antioxidant effects of PSE *in vivo* under normal physiological conditions was challenging, therefore, oxidative stress induced mouse models are recommended for validation.

Most plant extract studies often remain at the preclinical level, and the effects in clinical trials are often minimal. However, Clinical trials have been reported showing that when PSE was administered orally, hair density significantly increased and there were no side effects in the human body [33]. This dual basis further highlights the uniqueness and differentiation of PSE compared to other plant-derived ingredients studied in relation to hair growth.

Taken together, PSE promotes hair growth through a multifaceted mechanism that integrates Wnt/β-catenin activation, modulation of growth and inhibitory factors, and additional antioxidant/anti-inflammatory effects. This multimodal mechanism of action provides a novel perspective on the potential of botanical extracts to support hair follicle health and growth effectively.

Although direct toxicity evaluation was not performed in this study, the absence of cytotoxicity *in vitro* and the lack of reported adverse effects in clinical use suggest that PSE has potential for cosmetic and nutraceutical applications. Although some reports suggest that certain compounds found in peas such as isoflavones may exhibit estrogen-like activity [39]. Further research is warranted to determine the concentration of these compounds specifically in in pea sprouts and to evaluate their impact on human health at typical consumption levels. Therefore, establishing an optimal dosage threshold for PSE is essential to balance its efficacy with long-term safety.

Future studies are needed to clarify the detailed transcriptional mechanisms downstream of Wnt/β-catenin and to validate the antioxidant effects of PSE in vivo under physiological conditions.

In summary, this study demonstrates that pea sprouts extract (PSE) promotes hair growth through a multifactorial mechanism, involving activation of the Wnt/β-catenin pathway, upregulation of pro-anagen factors, and suppression of catagen-inducing signals. Additionally, its antioxidant and anti-inflammatory properties may further contribute to the protection of hair follicle. These findings suggest a comprehensive mechanism that may underlie its clinically observed efficacy. The product shows potential as a cosmetic or nutraceutical agent.

## Figures and Tables

**Table 1:** Primer sequence for qPCR.